# Bifurcated Dacron patch for simultaneous superficial femoroplasty and profundoplasty: a case report

**DOI:** 10.1186/1752-1947-3-9294

**Published:** 2009-11-24

**Authors:** Haris A Khwaja, Patrick OB Omotoso

**Affiliations:** 1 Bariatric and Metabolic Institute & Dept. of Surgery, Cleveland Clinic, 9500 Euclid Avenue, Cleveland 44950, Ohio, USA; 2 Bedfordshire Vascular Unit, Bedford Hospital NHS Trust, Bedford, South Wing, Kempston Road, Bedford, MK42 9DJ, UK

## Abstract

**Introduction:**

Common femoral endarterectomy and/or profundoplasty are procedures commonly performed on patients with functional or critical limb ischaemia.

**Case presentation:**

A 61-year-old Caucasian British man was referred to our unit with recent onset of severe left calf and thigh claudication and rest pain in his left foot. Magnetic resonance angiography revealed occlusive disease of the left common femoral artery, proximal superficial and profunda femoral arteries.

These findings were confirmed intraoperatively and an endarterectomy was subsequently performed from the left common femoral onto the proximal superficial femoral artery and then onto the proximal profunda femoris artery. The arteriotomy was closed with a Dacron patch and its distal end was bisected into two to patch the profunda femoris and superficial femoral arteries. The patient made an uneventful recovery with a full clinical improvement of his left leg.

**Conclusion:**

A Dacron patch that was bisected distally to make a bifurcated patch for simultaneous patching of the profunda femoris artery and the superficial femoral artery was used to treat our patient's occlusions. This technique has not been previously described in the published literature and we have found it easy to do with little time added to conventional operation.

## Introduction

Common femoral endarterectomy and/or profundoplasty are procedures commonly performed on patients with functional or critical limb ischaemia. Autogenous vein or artery, prosthetic patch, allografts or xenografts like bovine pericardial patches can be used in performing profundoplasty, depending on prevailing circumstances.

We describe here the use of a Dacron patch that was bisected distally to make a bifurcated patch for simultaneous patching of the profunda femoris artery (PFA) and superficial femoral artery (SFA) in a patient with occlusions of the PFA and proximal SFA. This technique has not been previously described in the published literature and we have found it easy to do with little time added to conventional operation.

## Case presentation

A 61-year-old Caucasian British gentleman with a five-year history of stable left calf claudication at 100 yards was referred to our unit with recent onset of incapacitating left calf and thigh claudication as well as sudden onset of rest pain in his left foot. His only risk factor was smoking. He had no other comorbidities and was taking aspirin and simvastatin. Clinically he had a weak left femoral pulse with absent distal pulses on his left leg. His left foot was cool to touch but there was no evidence of ulceration. He had all his right lower limb pulses palpable. Ankle Brachial Pressure Indices (ABPI) were 0.37 on the left and 1 on the right ankle.

**Figure 1:**
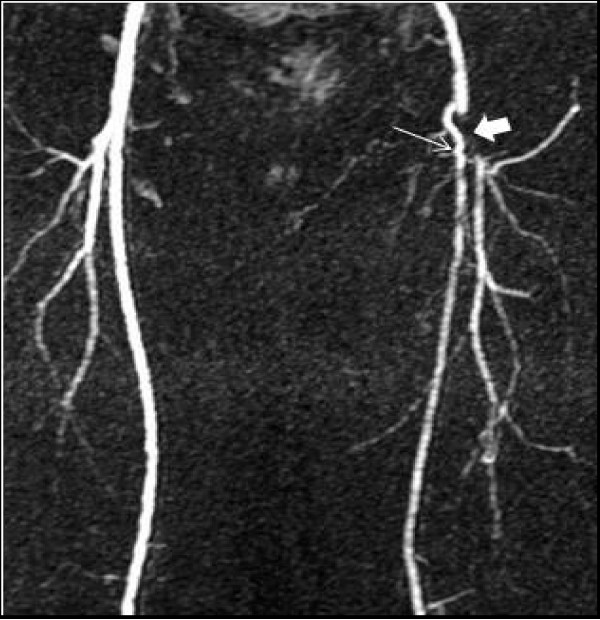
**A magnetic resonance angiography image demonstrating occluded left distal common and proximal superficial (thin arrow) and profunda (thick arrow) femoral arteries, with a collateral artery connecting the two**.

**Figure 2:**
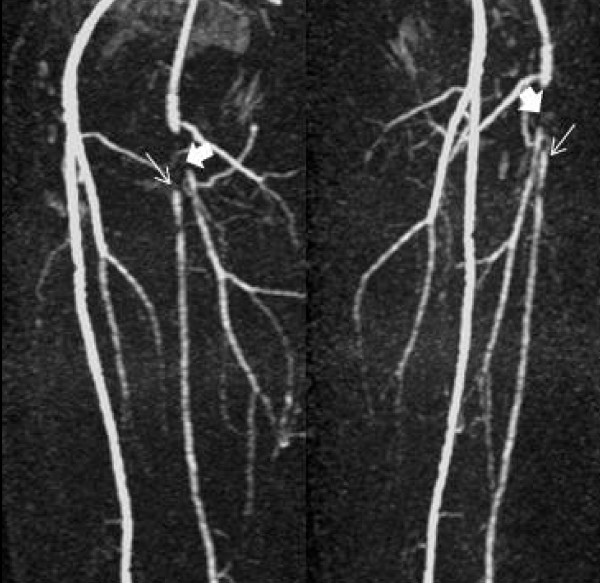
**Oblique magnetic resonance angiography images demonstrating the same lesions more clearly**.

Magnetic resonance angiography (MRA) revealed occlusive disease of the left common femoral artery (CFA) and of the proximal superficial and profunda femoral arteries (Figures 1, 2).

The patient underwent an endarterectomy and a prosthetic patch angioplasty of his left common, superficial and profunda femoral arteries. The main operative findings were occluded common, proximal superficial and profunda femoral arteries with sub-plaque hematoma. The occluding plaque was found at the bifurcation while the proximal SFA and PFA with a patent SFA and PFA were found beyond the plaque.

**Figure 3:**
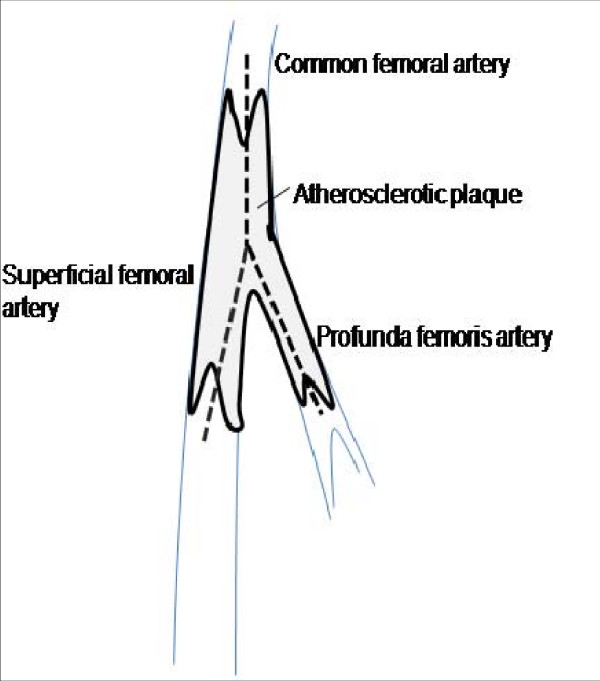
**Schematic diagram showing the common femoral, profunda femoris and superficial femoral arteries during arteriotomy**.

**Figure 4:**
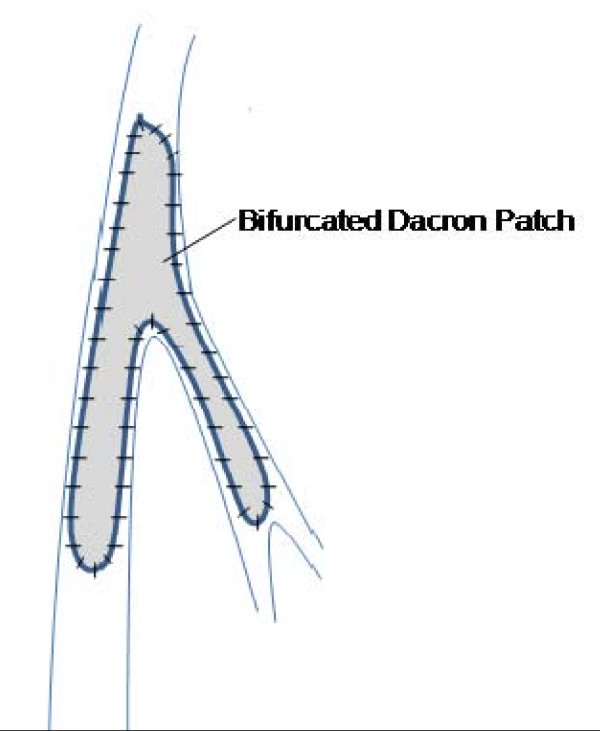
**Schematic diagram of intraoperative repair using the bifurcated Dacron patch**.

**Figure 5:**
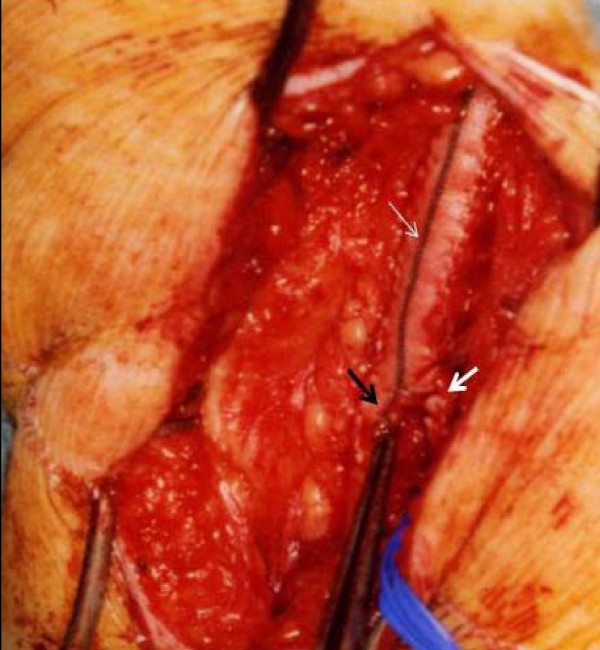
**An operative photograph demonstrating Dacron patch to common femoral artery (thin white arrow) and patch split at the distal end to simultaneously patch the profunda femoris (thick white arrow) and superficial femoral arteries (black arrow)**.

The endarterectomy was performed openly through an inverted Y arteriotomy extending from the left CFA onto the proximal SFA and then onto the proximal PFA (Figure 3). Tacking sutures of 7/0 prolene were applied to the distal end of the PFA and SFA. The arteriotomy was closed with a Dacron patch with its distal end bisected into two to patch the PFA and SFA simultaneously (schematic diagram of the surgical repair is shown in Figure 4 and the final intraoperative repair in Figure 5).

After the operation, the patient made an uneventful recovery with an improvement in his ABPI to 1.03 and a full resolution of the symptoms on his left leg.

## Discussion

Under normal circumstances, the PFA provides the main arterial supply to the thigh [1]. The PFA serves as the primary collateral channel from the iliac and common femoral arteries to the distal extremity [2]. Therefore, combined simultaneous diseases of the PFA and SFA lead to a worsening and aggravation of the degree of ischaemia. Our patient presented with a sudden shortening of his claudication distance and rest pain with an ABPI of 0.37 in his left lower limb on a background of chronic 100-yard claudication. We presumed that sub-plaque hemorrhage converted his previously stenotic disease to an occlusive one, which accounts for his sudden symptomatic deterioration. The simultaneous presence of diseases in the SFA and the PFA would explain his critical ischaemia in the face of a single level (i.e. femoral) arterial occlusive disease.

Because of the patient's rather friable plaque, we deemed it necessary to do an open SFA and PFA endarterectomy instead of a retrograde PFA endarterectomy through an SFA arteriotomy. As such we resorted to an inverted Y-shaped arteriotomy from the CFA onto the SFA and the PFA. This enabled precise endarterectomy and application of distal tacking sutures to prevent dissection. The usual presentation of critical limb ischaemia in patients with a degree of peripheral vascular disease this extensive makes the finding of significant occlusive disease in the SFA more common. An alternative to the Dacron graft that we chose to use would be bovine pericardium.

Autogenous vein patch angioplasty would have been ideal procedure, considering its advantages such as durability and a lower risk of infection. It is also possible to use the greater saphenous vein (GSV) with a large tributary as a bifurcated patch. However, in our patient, being a rather young arteriopath, we felt the need to preserve his GSV as it might be needed for cardiac or peripheral bypass in the future. As such we resorted to using a bifurcated patch of Dacron, which we created by hemisecting the patch distally along its marked midline.

We started the anastomosis distally in the SFA, the artery with a longer arteriotomy, with 5/0 prolene suture (after shaping the distal end of the patch accordingly). We ran the anastomosis proximally along the lateral side of the SFA and extended the patch bifurcation in small bits until the femoral bifurcation was reached. We then ran the anastomosis along the medial side of the PFA distally to the end of the arteriotomy, all the while shortening the patch as needed by the situation. The anastomosis was then completed circumferentially.

## Conclusion

A Dacron patch that was bisected distally to make a bifurcated patch for simultaneous patching of the profunda femoris artery and the superficial femoral artery was used to treat our patient's occlusions. This technique has not been previously described in the published literature and we have found it easy to do with little time added to conventional operation.

## Abbreviations

PFA: profunda femoris artery; SFA: superficial femoral artery; ABPI: Ankle Brachial Pressure Indices; MRA: magnetic resonance angiography; CFA: common femoral artery; GSV: greater saphenous vein.

## Competing interests

The authors declare that they have no competing interests.

## Authors' contributions

HAK analyzed and interpreted the patient data regarding the clinical presentation, imaging and writing of the manuscript. PO contributed to the writing of the Discussion section of the manuscript and performed the procedure described in the paper. All authors read and approved the final manuscript.

## Consent

Written informed consent was obtained from the patient for publication of this case report and any accompanying images. A copy of the written consent is available for review by the Editor-in-Chief of this journal.
